# Autophagy complexes cell death by necroptosis

**DOI:** 10.18632/oncotarget.10640

**Published:** 2016-07-16

**Authors:** Megan L. Goodall, Scott D. Cramer, Andrew Thorburn

**Affiliations:** Department of Pharmacology, University of Colorado Denver, Aurora, CO, USA

**Keywords:** Autophagy, apoptosis, necroptosis, cell death, Map3k7

Macroautophagy (autophagy) is a conserved cell survival pathway that catabolizes damaged proteins and organelles to maintain homeostasis and serves as a potential source of nutrients. Under basal cell conditions, cytosolic contents are sequestered by the autophagosome and ultimately delivered to the lysosome for degradation. The degradative process can in turn be upregulated in response to multiple stressors (e.g. starvation, therapeutics, oxidative stress) [1]. In addition to bulk non-specific turnover, several adaptor proteins, such as p62/SQSTM1, are known to recruit specific cargos to the autophagosome. The plasticity of autophagy in response to these differing stimuli and its ability to recruit and degrade selective cargo differentially modulates cell viability, enhancing both survival and death. An example of this is the autophagic degradation of FAP-1 (Fas Associated Phosphatase-1), a negative regulator of FAS, which enables FAS ligand induced apoptosis [2]. Although many studies have shown direct links between autophagy and apoptosis, very little is known connecting autophagy and other forms of programmed cell death, such as necroptosis [1]. Necroptosis, programmed necrosis, requires a cytosolic complex known as the necrosome. The necrosome is formed by the serine/threonine RIPK1 (receptor interacting protein kinase 1) in complex with RIPK3, FADD, and caspase-8 upon stimulation with TNF-α (tumor necrosis factor α). Upon trans- and autophosphorylation of RIPK1/RIPK3, MLKL (mixed kinase domain-like protein) is recruited, phosphorylated, and ultimately mediates plasma membrane permeablization inducing necroptosis [3].

**Figure 1:**
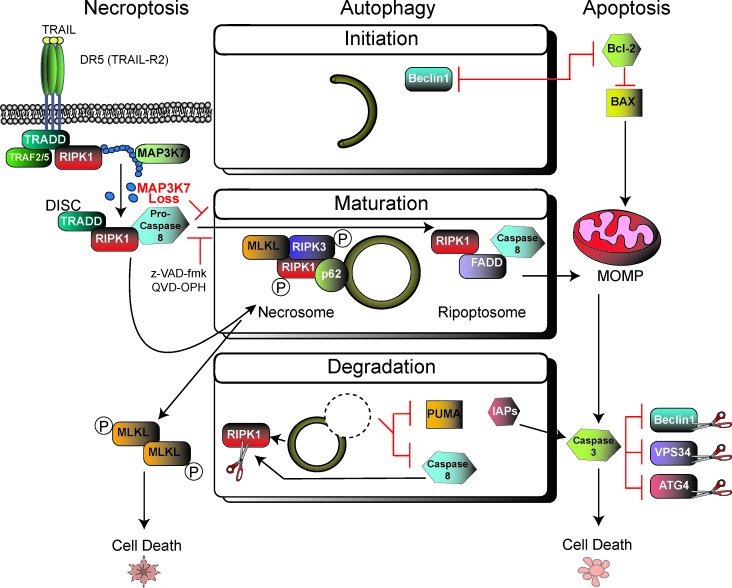
Crosstalk of autophagy with apoptosis and necroptosis The stages of autophagy are depicted in the middle; initiation, maturation (elongation), and degradation; with intersections of both the apoptotic and necroptotic pathways. On the left, binding of TRAIL to its receptor normally leads to formation of the DISC (Death-inducing Signaling Complex). However, loss of MAP3K7 (Mitogen-Activated Protein Kinase Kinase Kinase 7) leads to formation of the necrosome on the autophagosome and signals cell death through necroptosis. This is also accomplished if pan-caspase inhibitors z-VAD-fmk or QVD-OPH are applied. If the necrosome is prevented from forming, or signaling, apopotosis and downstream MOMP (Mitochondrial Outer Membrane Permeablization) can alternatively induce cell death. Numerous other known interactions with autophagy and apoptosis, on the right, are shown at each stage of the autophagy pathway. Protein abbreviations: TRADD (TNFRSF1A-Associated Via Death Domain), TRAF (TNF Receptor Associated Factors), p62/SQSTM1 (Sequestosome 1), Bcl-2 (B-cell lymphoma 2), BAX (Bcl-2 Associated X protein), VPS34 (Vacuolar Protein Sorting 34), ATG (Autophagy-related protein), Beclin1 (human orthologue of yeast ATG6), IAP (Inhibitors of Apoptosis Proteins), and PUMA (p53 Upregulated Modulator of Apoptosis).

Although a great deal has been reported about how autophagy and apoptosis modulate one another [4], the exact role of autophagy in cell death is still poorly understood. Moreover, autophagy's roles in cell death have primarily been studied in terms of its degradative properties (Figure 1). Our recent study highlighted a broader role for the autophagy machinery that is independent of its degradative functions by showing that the autophagy machinery serves as a scaffold for the necrosome complex formation, and ultimately its activation for signaling cell death through necroptosis [5]. In cells lacking *Map3k7*, a gene found to be lost in 30-40% of prostate cancers and associated with aggressive disease [6], we found a hypersensitivity to TNFα and TRAIL (TNFα-related apoptosis-inducing ligand)-induced cell death. Surprisingly however, despite the well-established mechanism of TRAIL being able to induce apoptosis, the *Map3k7*-null cells were shown to preferentially die through necroptosis. And, the cell death mechanism could switch to apoptosis when necroptosis was prevented by direct inhibition of the necrosome.

We have previously shown that TRAIL-induced apoptosis involves autophagy [7], therefore we further investigated the role of autophagy in necroptosis. Interestingly, inhibition of late stage autophagy enhanced cell death in response to TRAIL while inhibition of early/mid-stages of autophagy using genetic knockdown and pharmacological inhibition (ATG5, ATG7, Beclin 1, or Wortmannin) prevented cell death. These observations led us to hypothesize that components of the autophagy machinery were mediating the cell death by serving as a scaffold for efficient necrosome formation rather than by turnover of cellular components by autophagy.

In support of this hypothesis, localization of the necrosome complex to the autophagosome was confirmed through co-immunoprecipitations, dual proximity ligation assays, and immuno-gold transmission electron microscopy. Moreover, localization of the necrosome to the autophagosome machinery was shown to require p62/SQSTM1 binding to RIPK1 as loss of p62/SQSTM1 was sufficient to switch cell death from necroptosis to apoptosis. These data led us to conclude that p62-dependent recruitment of necrosome components to the autophagy machinery provides a mechanism for efficient activation of the necrosome, thus allowing the decision to be made for the cell to die by apoptosis versus necroptosis.

Overall, our studies provide critical insights into how autophagy regulates necroptosis and presents a mechanism by which controlled switching between necroptosis and apoptosis is accomplished. More importantly, our studies raise a number of significant issues: 1) autophagy machinery can play an important role in cell death regulation independent of the degradative pathway, and 2) It is critical to take into consideration at which point the autophagy pathway is inhibited, not just if the pathway is inhibited. Indeed, inhibition at different stages of the autophagy pathway could sometimes have opposing outcomes.
